# Somatostatin and its 2A receptor in dorsal root ganglia and dorsal horn of mouse and human: expression, trafficking and possible role in pain

**DOI:** 10.1186/1744-8069-10-12

**Published:** 2014-02-13

**Authors:** Tie-Jun Sten Shi, Qiong Xiang, Ming-Dong Zhang, Swapnali Barde, Ylva Kai-Larsen, Kaj Fried, Anna Josephson, Laura Glück, Sergey M Deyev, Andrei V Zvyagin, Stefan Schulz, Tomas Hökfelt

**Affiliations:** 1 Department of Neuroscience, Karolinska Institutet, Stockholm, Sweden; 2 School of Life Science and Technology, Harbin Institute of Technology, Harbin, China; 3 Department of Dental Medicine, Karolinska Institutet, Stockholm, Sweden; 4 Department of Medical Biochemistry and Biophysics, Karolinska Institutet, Stockholm 17177, Sweden; 5 Department of Pharmacology and Toxicology, Jena University Hospital, Friedrich Schiller University, Jena, Germany; 6 Shemyakin & Ovchinnikov Institute of Bioorganic Chemistry, Moscow, Russia; 7 MQ Biofocus Research Centre, Macquarie University, Sydney, NSW, Australia

**Keywords:** Axonal transport, Nerve injury, Neuropeptide, NPY receptor, Receptor internalization, Retrograde signaling

## Abstract

**Background:**

Somatostatin (SST) and some of its receptor subtypes have been implicated in pain signaling at the spinal level. In this study we have investigated the role of SST and its sst2A receptor (sst2A) in dorsal root ganglia (DRGs) and spinal cord.

**Results:**

SST and sst2A protein and sst2 transcript were found in both mouse and human DRGs, sst2A-immunoreactive (IR) cell bodies and processes in lamina II in mouse and human spinal dorsal horn, and sst2A-IR nerve terminals in mouse skin. The receptor protein was associated with the cell membrane. Following peripheral nerve injury sst2A-like immunoreactivity (LI) was decreased, and SST-LI increased in DRGs. sst2A-LI accumulated on the proximal and, more strongly, on the distal side of a sciatic nerve ligation. Fluorescence-labeled SST administered to a hind paw was internalized and retrogradely transported, indicating that a SST-sst2A complex may represent a retrograde signal. Internalization of sst2A was seen in DRG neurons after systemic treatment with the sst2 agonist octreotide (Oct), and in dorsal horn and DRG neurons after intrathecal administration. Some DRG neurons co-expressed sst2A and the neuropeptide Y Y1 receptor on the cell membrane, and systemic Oct caused co-internalization, hypothetically a sign of receptor heterodimerization. Oct treatment attenuated the reduction of pain threshold in a neuropathic pain model, in parallel suppressing the activation of p38 MAPK in the DRGs

**Conclusions:**

The findings highlight a significant and complex role of the SST system in pain signaling. The fact that the sst2A system is found also in human DRGs and spinal cord, suggests that sst2A may represent a potential pharmacologic target for treatment of neuropathic pain.

## Background

Somatostatin (SST) is a regulatory peptide produced by neurons and many other cell types [1-3]. Originally discovered as the hypothalamic growth hormone release-inhibiting hormone [4], SST was rapidly found to have a wide extrahypothalamic distribution, also in peripheral neural and non-neural tissues [5,6]. An early study demonstrated a depressant action of SST on brain neurons [7], confirmed in many studies: SST always exerts inhibition.

SST was found in small dorsal root ganglion (DRG) neurons [8,9], many of which weakly express pre-protachykinin A [10] and most of them calcitonin gene-related peptide (CGRP) [10,11]. A dense SST-positive^(+)^ fiber plexus in the superficial dorsal horn mainly originates from small local interneurons [12-14]. Electrophysiological studies have shown that SST exerts an inhibitory effect on dorsal horn neurons [15-19]. Moreover, SST and its analogues have anti-nociceptive and anti-inflammatory effects in experimental animals [15,20-31], and relieve pain in humans [32-34].

SST interacts with five receptor subtypes, sst1-5 [35-38]. Autoradiography demonstrated I^125^-binding over the dorsal horn [39], a pattern also seen with a sst2-selective ligand [40]. Two variant forms, sst2A and sst2B generated by alternative splicing, have been identified in DRG neurons [24,41-44], sst2B having a different localization [43]. Other SSTR subtypes are also found in DRGs [24,41,45,46]. The inhibitory effects of SST on primary sensory neurons are at least partly exerted via sst2A [24,45,47-50].

Here we analyzed the expression of sst2A and SST at the spinal level in mouse, and some aspects also in human tissues. The axonal transport of sst2A and SST was studied after sciatic nerve ligation and after administration of fluorescence-labeled SST into the paw. Two neuropeptide Y (NPY) receptor subtypes were included for comparison. Receptor trafficking was studied after systemic and intrathecal (ith) administration of the sst2 agonist octreotide (Oct). In addition we monitored pain thresholds in the spared nerve injury (SNI) model [51,52] in wild type (WT) and sst2 knock-out (KO) mice.

## Results

### Expression of sst2A in mouse DRGs

**Figure 1:**
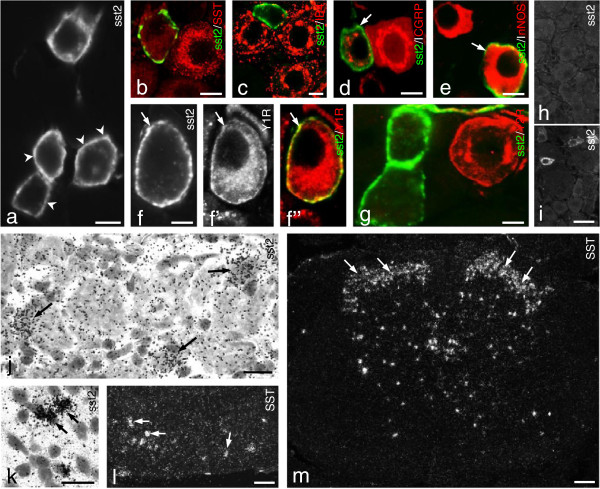
**sst2A-LI in mouse DRGs. (a)** Several sst2A^+^ neurons are seen, and receptor protein is mainly located along the somatic plasmalemma (arrowheads). **(b-e, f”,g)** Color images show merged micrographs after double-staining (**f-f”** show the same section). **(d-f”)** Arrows indicate the coexistence of sst2A with CGRP **(d)**, nNOS **(e)** and Y1R **(f-f”)**, respectively. **(b, c, g)** sst2A-LI cannot be detected in SST^+^**(b)**, IB4^+^**(c)** or Y2R^+^**(g)** neurons. CGRP-and nNOS-LIs are mainly seen in the perinuclear region **(d, e)**, while Y1R-LI is found both in the plasmalemma and in the perinuclear region **(f’, f”)**. **(h, i)** Lack of sst2A-LI in DRGs of sst2-KO mouse **(h)** but presence in WT mouse **(i)**. **(j, k)** Expression of sst2 mRNA in DRG neurons (**j**, arrows) and spinal dorsal horn neurons (**k**, arrows). **(l, m)** Expression of SST mRNA in DRG neurons (l, arrows) and spinal dorsal horn (**m**, arrows). Scale bars indicate 10 μm **(a-g, k)**, 20 μm **(j)**, 50 μm **(i, l)** and 100 μm **(m)**.

The immunohistochemical analysis, in general, showed that sst2A-like immunoreactivity (LI) was concentrated along the neuronal cell membrane, with only minor staining in the cytoplasm (Figure 1a). In control lumbar 5 (L5) DRGs, around 12% of all neuron profiles (NPs) were sst2A^+^ with a size distribution ranging from 100 to 1,200 μm^2^, the majority between 200 and 600 μm^2^, mainly representing small-sized NPs [53]. This is essentially in agreement with the situation in mouse [24], and similar to rat [43,44,46]. No sst2A^+^ NPs expressed SST (Figure 1b), as previously reported for rat [44], that is sst2 does not represent an autoreceptor. Almost no sst2A^+^ NPs were isolectin B4 (IB4)^+^ (~1%; Figure 1c), a marker for non-peptidergic neurons. In contrast, most sst2A^+^ NPs (98.6 ± 1.0%) expressed CGRP (Figure 1d), that is they belong to the peptidergic population [54]. About one fifth (17.9 ± 4.6%) of the sst2A^+^ NPs was neuronal nitric oxide synthase (nNOS)^+^ (Figure 1e). Some sst2A^+^ neurons contained neuropeptide Y receptor subtype 1 (Y1R) (Figure 1f-f”), but not subtype 2 (Y2R) (Figure 1g). sst2A- LI was completely absent in the DRGs (Figure 1h vs. i) of sst2-KO mice. Both sst2 and SST mRNA^+^ neurons were seen in DRGs with *in situ* hybridization, providing confirmation at the transcript level (sst2, Figure 1j; SST, Figure 1l).

### Expression of sst2A in mouse dorsal horn

**Figure 2:**
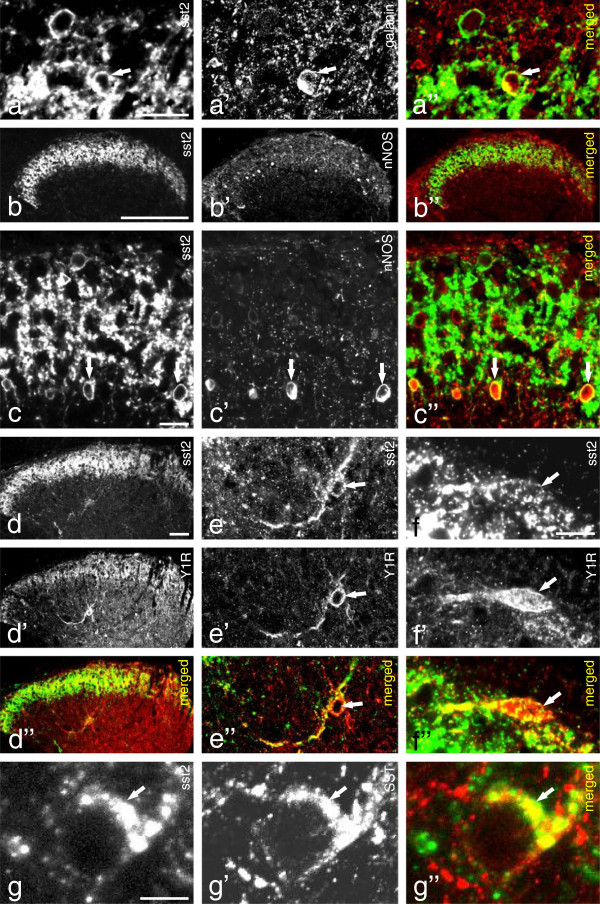
**sst2A-LI in mouse dorsal horn. (a, b, c, d)** sst2A-LI is expressed in a dense plexus of processes in superficial layers in spinal dorsal horn (L4–5 segments) and in cell bodies in lamina I, II (many) and lamina IV (few). **(a-a”)** A few of the sst2A^+^ local neurons, mainly in the inner layer of lamina II, are galanin^+^ (arrows). **(b-b”, c-c”)** Many sst2A^+^ neurons express nNOS-LI in outer layer of lamina II (arrows). **(d-f”)** sst2A^+^ neurons are Y1R^+^ both in deeper layers (lamina IV; **d-d”** and **e-e”**; arrows) and superficial layers (**f-f”**; arrows). A few sst2A-IR neurons in lamina II are also SST^+^ (**g-g”**, arrows). Scale bars indicate 2 μm **(g)**, 5 μm **(f)**, 10 μm **(a, c)**, 50 μm **(d)** and 200 μm **(b)**.

**Figure 3:**
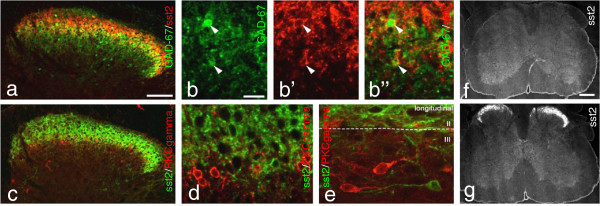
**sst2A-LI within dorsal horn of a GAD-67-GFP knock-in mouse. (a-e)** sst2A^+^ neurons are co-localized with GAD-67-GFP (b-b”, arrowheads), but not with PKC gamma **(d, e)**. **(f, g)** sst2A-LI cannot be detected in sst2-KO mouse **(f)** as in WT mouse **(g)**. Scale bars indicate 20 μm **(b)**, 100 μm **(a)** and 200 μm **(f)**.

sst2A-LI was observed in a dense fiber plexus in the superficial layers in spinal dorsal horn of the L4–5 segments (Figure 2a, b, c, d), with many intermingled sst2A-immunoreactive (IR) cell bodies (Figure 2a, c). A few of the sst2A^+^ local neurons, mainly in the inner layer of lamina II, were galanin^+^ (Figure 2a-a”). Many sst2A^+^ neurons co-expressed nNOS in outer layer of lamina II (Figure 2b-c”). Few sst2A^+^ neurons were Y1R^+^ both in superficial lamina I (Figure 2f-f”) and in the deeper layers, such as lamina IV (Figure 2d-d” and e-e”). A small number of sstA2^+^ neurons were SST^+^ in lamina II (Figure 2g-g”). Furthermore, in the spinal cord of GAD-67-GFP transgenic mice, the sst2A^+^ fibers were found to overlap with GAD-67-GFP^+^ fibers in the superficial layers, mainly in lamina II (Figure 3a). Most sst2A^+^ interneurons were GAD-67-GFP^+^ (Figure 3b-b”), but none PKCgamma^+^ (Figure 3c-e), a marker for excitatory interneurons [55]. Some spinal dorsal horn neurons expressed transcript for sst2 (Figure 1k) or SST (Figure 1m). sst2A-LI was not detected in the spinal cord of sst2-KO mice (Figure 3f vs. g).

### Expression of sst2A in human DRG and dorsal horn

**Figure 4:**
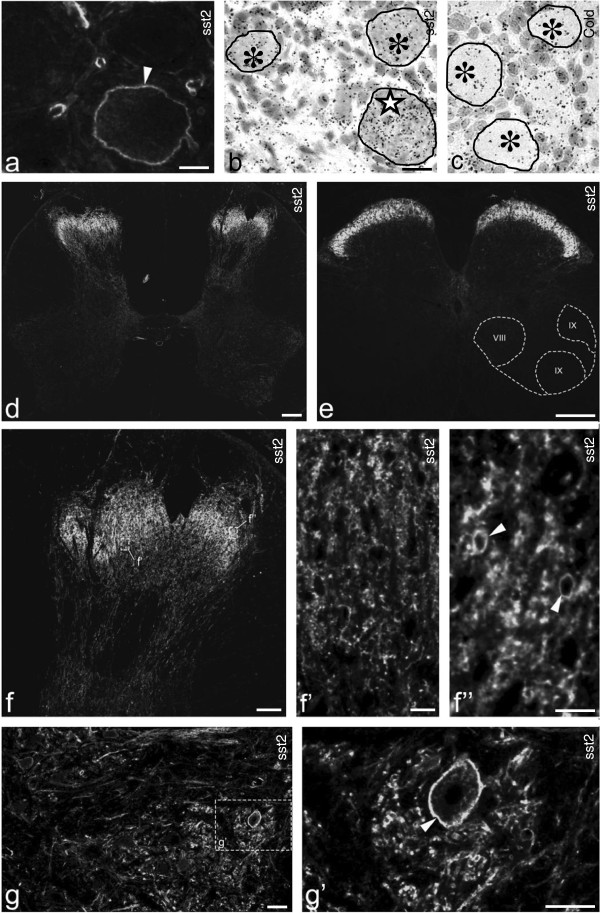
**sst2A in human DRG and spinal cord. (a)** DRG section from human. sst2A-LI is detected in very few neurons. The immunoreactivity is associated with the cell membrane (arrowheads). **(b-c)**. sst2 mRNA is expressed in human DRG incubated with antisense probe (**b**; white star, sst2^+^; black asterisks, sst2^-^) but not with cold probe (**c**; black asterisks, sst2^-^). **(d-g’)** sst2A-LI is seen throughout the gray matter in human spinal cord with a gradient from dorsal to ventral horn **(d)**, whereas in mouse the sst2A-LI is mainly present in lamina I and II, with some staining in lamina X and the ventral horn **(e)**. **(f-g’)** Strong sst2A-LI is observed mainly in superficial layers with a modest density in deeper laminae **(f)**. High magnifications show a dense plexus of sst2A^+^ processes (**f’**; box in **f**) and some sst2A^+^ interneurons in the dorsal horn (**f”**; arrowheads; box in f). sst2A^+^ terminals and motor neurons are also observed in the ventral horn **(g, g’)**. Arrowhead indicates a motoneuron with strong membrane and relatively weak cytoplasmic sst2A-LI (g’; box in g). Scale bars indicate 20 μm **(a, b, f”)**, 50 μm **(f’, g, g’)**, 250 μm **(e, f)** and 500 μm **(d)**.

sst2A-LI was observed in a few human DRG neurons, that is only single NPs per section. The immunoreactivity was associated with the cell surface membrane (Figure 4a). A small population of neuronal cells expressed notable levels of sst2 mRNA in human DRGs (Figure 4b). In human spinal cord, sst2A staining was observed in all layers of the grey substance (Figure 4d), that is more widely distributed than in mouse (Figure 4d vs. e). The highest density was seen in lamina II (Figure 4d,f), where both processes (Figure 4f’) and cell bodies (Figure 4f”) were stained. The remaining layers had modest densities of fibers (Figure 4d). In the ventral horns some motoneuron membranes were distinctly labeled and often surrounded by many punctuate, sometimes ring-formed structures (Figure 4g,g’). The latter may represent dendrites ‘decorated’ by sst2A receptors.

### Axonal transport of sst2A

**Figure 5:**
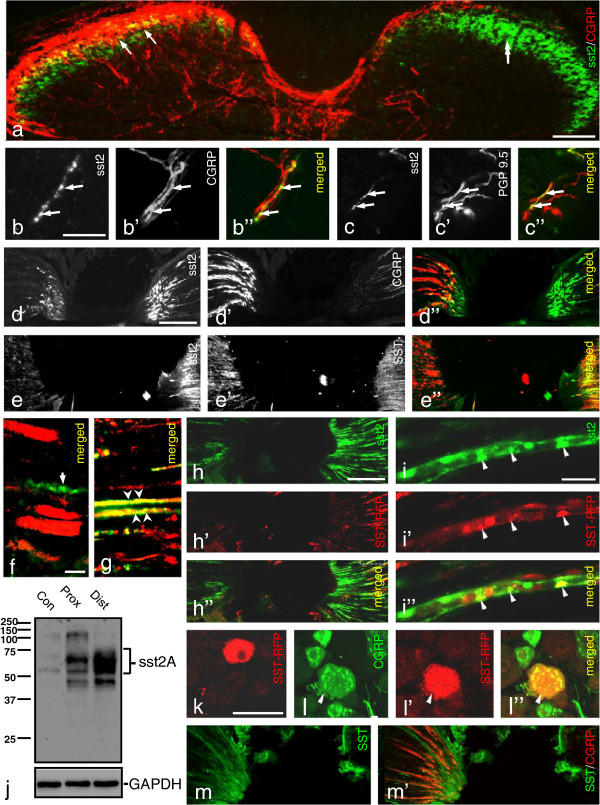
**Axonal transport of sst2A-LI. (a)** Double-staining shows strong expression of sst2A-LI in the superficial dorsal horn, partially overlapping with the CGRP-LI (arrows in **a**). Fourteen days after unilateral dorsal rhizotomy, there is a dramatic reduction of CGRP-LI ipsilaterally (double-arrow in **a**) but not contralaterally (arrows in **a**). sst2A-LI seems unaffected. **(b-c”)** Skin of control mouse hindpaw. A few peripheral terminals of sciatic nerve contain sst2A-LI that co-localizes with CGRP (arrows in **b-b”**, same section) or PGP9.5 (arrows in **c-c”**, same section). **(d-d”)** A 10-hr ligation causes a modest accumulation of sst2A-LI on the proximal side, but a much stronger one distally (right), versus the mainly proximal CGRP pile-up (left) (cf. **d’** with **d**; **d”)**. **(e-e”)** SST-LI is present on both sides of the lesion **(e’)**, and highly coexpressed with sst2A on the distal, but not on the proximal side (right) (cf. **e’** with **e**; **e**”). Double-staining for sst2A and SST indicates possible coexistence on the distal (**g**; arrowheads) but not proximal side (**f**; an arrow points to an sst2A^+^ fiber lacking SST-LI). **(j)** Western blot showing higher sst2A protein levels on the distal than on the proximal side 10 hrs after ligation. **(h-h”)** Accumulation of sst2A and SST-RFP on the distal side after SST-RFP injection into the hind paw. **(i-i”)** The SST-RFP conjugate in distal axons is co-localized with sst2A (arrowheads). **(k-l”)** A few SST-RFP^+^ neurons are seen in ipsilateral DRGs 3 days after injection **(k)**, most being CGRP^+^**(l-l”)**. **(m, m’)** In sst2 KO mouse SST-LI only accumulates on the proximal side (contrasting the WT mouse, e’). CGRP-LI accumulates on the proximal side as see in WT mice **(m’)**. Scale bars indicate 10 μm **(f)**, 20 μm **(i)**, 50 μm **(k)**, 100 μm **(a, b, d)** and 200 μm **(h)**.

A 14-day dorsal rhizotomy did apparently not eliminate sst2A-LI in the ipsilateral dorsal horn, contrasting the virtually complete depletion of CGRP-LI (Figure 5a). However, it is likely that disappearance of a low number of sst2A^+^ sensory afferents cannot be detected in view of the very dense network of local sst2A^+^ processes and cell bodies. In the glabrous skin of the hind paw, sst2A-, CGRP-LIs (Figure 5b-b”) and PGP9.5 (Figure 5c-c”) apparently colocalize in fibers at the epidermal-dermal junction.

Ten hours after a nerve crush, sst2A-LI had accumulated around the lesion, but mainly on the distal side (Figure 5d,d”,e,e”,g,j). In contrast, strong CGRP-LI was seen on the proximal side (Figure 5d’,d”). SST-LI was present on both sides of the lesion (Figure 5e’,e”), and apparently coexpressed sst2A-LI on the distal side (Figure 5e-e”, g vs. f).

sst2A/CGRP coexistence in axonal swellings appeared limited at the crush site, perhaps surprising in view of the almost total sst2A/CGRP coexistence in DRG cell bodies. This may, however, be due to the fact that CGRP is expressed in a large proportion of the DRG neurons (>50%) [56], versus presence of sst2A in just 12%. Thus, the weak sst2A accumulation, together with the CGRP abundance in non-sst2A^+^ axons made it difficult to establish sst2A and CGRP coexistence in axons proximal to the crush. In agreement with the immunohistochemical findings, western blot results showed a higher sst2A protein level distally than proximally (Figure 5j).

Both sst2A and SST-RFP-LIs were detected on the distal side of the sciatic nerve 10 hrs after ligation combined with unilateral injection of the SST-RFP conjugate into the hind paw (Figure 5h-h”), the SST-RFP apparently being co-localized with sst2A (Figure 5i-i”). A few SST-RFP^+^ neurons were also seen in the ipsilateral DRGs 3 days after injection (Figure 5k), and most of them were CGRP^+^ (Figure 5l-l”). As seen in the WT mouse, CGRP-LI accumulated on the proximal side of the ligation in the sst2-KO mouse (Figure 5m’). However, in the sst2A-KO mouse SST-LI was mostly seen on the proximal side with much lower levels on the distal side of the ligation (Figure 5m), that is distinctly different from the control mouse.

### sst2A trafficking

**Figure 6:**
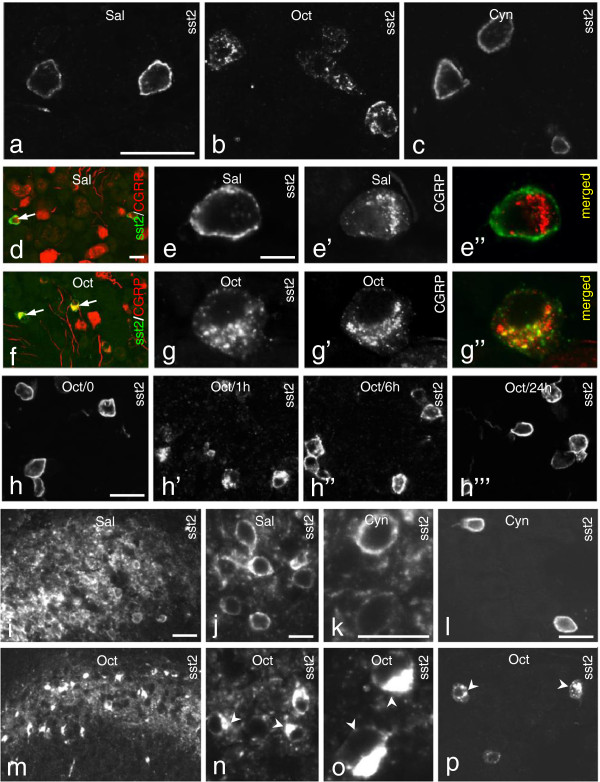
**Trafficking of sst2A-LI induced by Oct treatment. (a-g”)** One hr after Oct treatment a strong sst2A-LI is observed in the perinuclear region of the DRG neurons **(b)**, versus plasmalemma localization after saline **(a)** or Cyn154806 **(c)**. **(d-g”)** sst2A^+^ and CGRP^+^ neurons before and after Oct treatment (arrows in **d** and **f**, respectively). In controls sst2A-LI is mainly located on the plasma membrane and CGRP-LI in the perinuclear region **(e-e”)**. After Oct treatment, both CGRP-and sst2A-LI are detected in the perinuclear region **(g-g”)**. **(h-h”’)** Internalization of sst2A-LI in DRG neurons before **(h)** or 1 hr **(h’)**, 6 hrs **(h”)** or 24 hrs **(h”’)** after Oct administration. **(i-p)** Internalization of sst2A-LI in the dorsal horn neurons (lamina I-II) 1 hr after ith injection of Oct **(m, n, o)**, not seen after saline **(i, j)** or Cyn154806 **(k)**. Ith Oct **(p)**, but not Cyn154806 **(l)**, induces internalization of sst2A-LI in DRG neurons. Arrowheads in **n**, **o** and **p** indicate neurons with internalized sst2A. Scale bars indicate 10 μm **(j, k)**, 15 μm **(e)**, 25 μm **(i)** and 50 μm **(a, d, h, l)**.

We analyzed the effect of Oct, a well-known SST agonist with high affinity for sst2 [57,58] and of Cyn154806, potent and selective sst2 antagonist [50,59,60]. Systemic (i.v) administration of Oct (1 μg/10 μl) induced sst2A translocation in DRG neurons. Thus, after 1 hr a strong sst2A-LI was present in the perinuclear region (Figure 6b), whereas sst2A-LI was predominantly associated with the somatic plasmalemma in both saline (Figure 6a) and antagonist (Cyn154806; 0.2 μg/10 μl) (Figure 6c)-treated groups. Double-staining showed that almost all sst2A^+^ NPs contained CGRP-LI before (Figure 6d) and after Oct treatment (Figure 6f), the sst2A-LI mainly located on the plasma membrane, and the CGRP-LI in the perinuclear region (Figure 6e-e”). After Oct treatment, sst2A-LI was strongly expressed in the perinuclear region overlapping with CGRP (Figure 6g-g”). The internalized receptor was partly back to the membrane after 6 hrs and virtually fully back after 24 hrs (Figure 6h-h”’).

Internalization of sst2A-LI was also observed in dorsal horn neurons, mainly in laminae I-II, 1 hr after ith injection of Oct (1 μg/10 μl; Figure 6m,n,o), neither seen after saline (Figure 6i,j) nor Cyn154806 (0.2 μg/10 μl; Figure 6k). Furthermore, central administration of Oct also induced internalization of sst2A-LI in the DRG neurons (Figure 6p), not seen after saline (data not shown) or Cyn154806 (Figure 6l).

### Are sst2A and Y1R heterodimerized in mouse DRG neurons?

**Figure 7:**
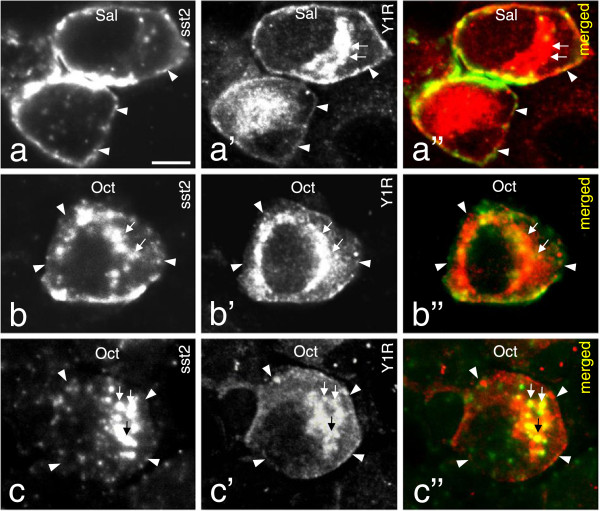
**Systemic administration of Oct induces internalization of both sst2A and Y1R. (a-a”)** sst2A and Y1R co-localized at the cell surface in control DRGs. (**b-b”** and **c-c”**; show two examples). One hr after Oct treatment sst2A **(b, b”, c, c”)** and Y1R **(b’, b”, c’ ,c”)**-LIs are mainly seen in the cytoplasm, partly overlapping. Note that Y1R still can be seen on the membrane **(c”)**. Arrowheads indicate membrane and arrows cytoplasmic staining. Scale bar indicates 10 μm **(a)**.

Double-labeling of sst2A and Y1R was observed in some DRG neurons (Figures 1f-f”, 7a-a”). It is known that the Y1R is in rat DRGs is present in a high percentage of the CGRP^+^ NPs, also this receptor mostly membrane-associated [61,62]. After Oct application (1 μg/10 μl) not only sst2A was internalized but also the Y1R. Thus, the distribution of both receptors decreased at the cell-surface, with a parallel increase in intracellular levels and with partly overlapping localization (Figure 7b-b” and c-c”). This process, and the overlap in the plasma membrane, suggests a possible heterodimerization.

### sst2A expression after nerve injury

**Figure 8:**
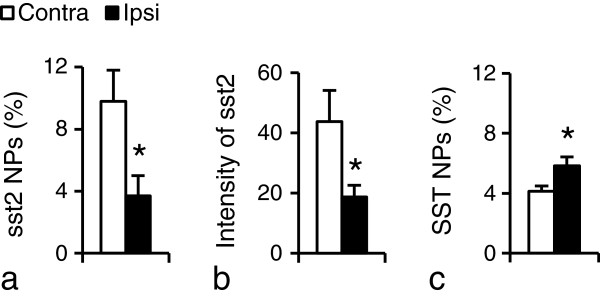
**sst2A-LI after SNI. (a, b)** Percentage of sst2A^+^NPs **(a)** and sst2A protein levels (fluorescence intensity) **(b)** are significantly decreased 14 days after SNI. **(c)** A slight but statistically significant increase in percentage of SST ^+^NPs is seen in the ipsilateral DRGs compared to the contralateral ones. *P < 0.05, compared with contralateral DRGs; n = 4 or 6 in each group.

Fourteen days after SNI a significant downregulation of sst2A-LI was observed in the ipsilateral DRGs, both with regard to percentage of NPs (Figure 8a; 4.0 ± 1.0% vs. 10.0 ± 1.0%; p < 0.05; n = 6/group) and fluorescence intensity (Figure 8b; 18.7 ± 3.9 vs. 43.7 ± 10.3; p < 0.05; n = 4/group). However, no difference in size distribution of sst2A^+^ NPs was found between ipsi- and contralateral DRGs (data not shown). A slight, but statistically significant increase of SST was seen in the ipsilateral DRGs (Figure 8c; 5.83 ± 0.6% vs. 4.1 ± 0.4%; p < 0.05; n = 6/group). Moreover, in contrast to the upregulation of sst1 (2.0 ± 0.3 vs. 1.0 ± 0.4; p < 0.01; n = 5/group), sst2 mRNA levels were significantly reduced in the ipsilateral DRGs, when compared with controls after a 2-week axotomy (0.6 ± 0.1 vs. 1.0 ± 0.2; p < 0.05; n = 4/group). Neither sst2 (1.0 ± 0.1 vs. 1.0 ± 0.1; p > 0.05; n = 5/group) nor sst1 (1.0 ± 0.1 vs. 1.0 ± 0.1; p > 0.05; n = 4/group) mRNA levels in the spinal cord were affected by nerve injury, respectively.

### Oct attenuates SNI-induced hyperalgesia

**Figure 9:**
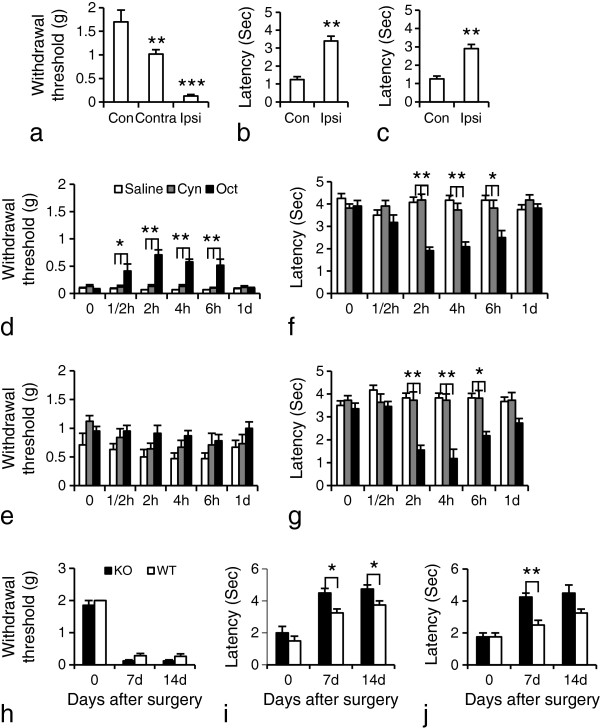
**Oct increases pain threshold 14 days after SNI. (a)** The mechanical threshold is reduced mainly in the ipsilateral hindpaw (n = 12), as compared with controls (n = 10). **(b, c)** The withdrawal response duration after cold stimulation **(b)** or nociceptive mechanical stimulation **(c)** is significantly increased ipsilaterally. Data are expressed as mean ± SEM. **P < 0.01 or ***P < 0.001 compared with the control group, respectively (n = 8 or 10 in each group). **(d)** Oct causes an ipsilateral increase in mechanical threshold as compared with saline- or Cyn154806 treatment (n = 11 or 12 in each group). **(e)** A transient effect of Oct on mechanical threshold is also found contralaterally, but does not reach significance. **(f, g)** Cold and Pin-prick tests in ipsilateral paws. A transient but pronounced effect is detected in Oct-treated group compared to saline- or Cyn154806-treated groups, most pronounced between 2 and 6 hrs. Data are expressed as mean ± SEM. *P < 0.05; **P < 0.01 compared with the saline- or Cyn154806-treated groups, respectively (n = 11 or 12 in each group). **(h)** After SNI injury, both sst2-KO mice and WT mice develop a significant ipsilateral reduction in the mechanical threshold as compared with baseline values. **(i, j)** Withdrawal response duration for both cold allodynia **(i)** and Pin-prick hyperalgesia **(j)** is significantly increased in both KO and WT mice on day 7 and 14. The effects are more pronounced in KO mice than in WT mice (n = 4 in each group). Data are expressed as mean ± SEM. *P < 0.05; **P < 0.01 compared with WT mice; n = 4 in each group.

Fourteen days after SNI, mice developed mechanical allodynia-like behavior as shown by the decrease in withdrawal threshold of the hindpaw ipsilateral to the nerve injury, also seen contralaterally, but less pronounced (Figure 9a; Con vs. Ipsi, 1.7 ± 0.3 vs. 0.1 ± 0.03, p < 0.001; Contra vs. Ipsi, 1.0 ± 0.1 vs. 0.1 ± 0.03, p < 0.01; n = 10 or 12/group). Latencies after both acetone (cold stimulation) and Pin-prick (noxious mechanical stimulation) tests were significantly increased (Figure 9b, Con vs. Ipsi, 1.3 ± 0.2 vs. 3.4 ± 0.3, p < 0.01; c, Con vs. Ipsi, 1.3 ± 0.2 vs. 2.9 ± 0.2, p < 0.01; n = 8 or 10/group).

Gross examination revealed that neither Oct (40 μg/kg, i.p.) nor Cyn154806 (Cyn; 6 mg/kg, i.p.) induces any obvious sedation or impaired motor function as compared with saline-treated (vehicle) animals. When given at day 14 after SNI, Oct significantly increased ipsilateral withdrawal threshold already after 30 min (0.4 ± 0.1 vs. 0.1 ± 0.01; p < 0.05, compared with vehicle), as monitored with mechanical stimulation with von Frey hairs, an effect still observed after 6 hrs (0.5 ± 0.1 vs. 0.1 ± 0; p < 0.01, compared with vehicle; n = 11 or 12/group), but returning to vehicle levels at 24 hrs (0.1 ± 0.01 vs. 0.1 ± 0.01; p > 0.05, compared with vehicle; n = 11 or 12/group) (Figure 9d). A trend, but not statistically significant effect, was seen contralaterally from 30 min to 24 hrs (Figure 9e). A significant reduction of the withdrawal response duration induced by acetone or Pin-prick was seen between 2 and 6 hrs in Oct-treated animals in both tests (Figure 9f,g). Treatment with the antagonist alone did not influence the pain threshold (Figure 9d-g; n = 11 or 12/group).

Deletion of sst2A did not affect the mechanical or cold threshold in mutant mice when compared with WT mice (Figure 9h-j). SNI induced mechanical allodynia in both KO and WT mice in the ipsilateral hindpaw post-operation days 7 and 14 (Figure 9h; n = 4/group). An increased withdrawal response duration was observed in both KO and WT mice in cold (Figure 9i) and Pin-prick (Figure 9j) tests at day 7 and 14 after operation, but it was more pronounced in the KO mice than in the WT mice (Figure 9i, p < 0.05; n = 4/group; Figure 9j; 4.3 ± 0.3 vs. 2.5 ± 0.3, p < 0.01; n = 4/group), suggesting that the sst2A exerts a weak protection against these two types of pain.

### Oct attenuates nerve injury-induced p-p38 upregulation after nerve injury

**Figure 10:**
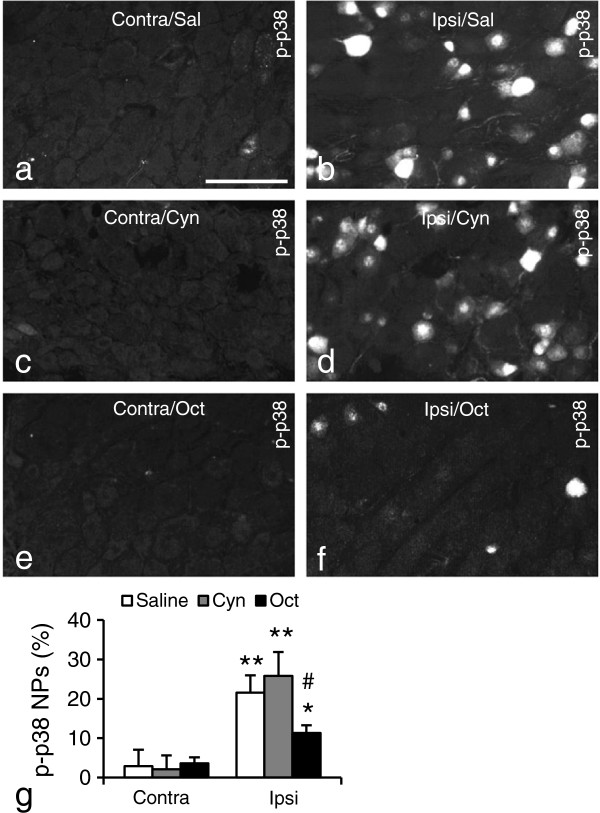
**Effect of Oct on p-p38 expression DRGs after SNI. (a-f)** Micrographs of contralateral **(a, c, e)** or ipsilateral DRGs **(b, d, f)**, in saline (Sal; **a, b**), Cyn154806 (Cyn; **c, d**) or Oct **(e, f)** -treated animals 14 days after SNI. **(g)** Quantitative evaluation of percentage of p-p38^+^ NPs. Oct, but not Cyn154806 or saline significantly reduces the SNI-induced ipsilateral upregulation of p-p38-LI. *P < 0.05; **P < 0.01 compared with contralateral DRGs; ^#^P < 0.05 compared with saline or Cyn154805 groups in the ipsilateral DRGs, respectively. Scale bar indicates 100 μm **(a)**.

p-p38 [63,64], is an important downstream substrate of the SST/sst2 signaling pathway [65-68]. Fourteen days after SNI p-p38 levels were analyzed in DRG neurons after 1 hr treatment (i.p. injection) with Oct, Cyn154806 or saline. SNI induced a significant upregulation of p-p38^+^ NPs in the ipsilateral DRGs, both in saline- and Cyn154806-treated groups (Figure 10b vs. a, 21.6 ± 4.4% vs. 2.9 ± 4.2%, p < 0.01; d vs. c, 25.8 ± 6.1% vs. 2.1 ± 3.5%, p < 0.01; g; compared with contralateral, respectively; n = 5/group). However, this increase was much less pronounced in Oct-treated animals (Figure 10f vs. e; g; 11.3 ± 2.0% vs 3.6 ± 1.5%, p < 0.05; compared with contralateral; p < 0.05, compared with saline or Cyn154806-treated animals in the ipsilateral DRGs, respectively; n = 5/group). The strong ipsilateral p-p38 upregulation, paralleling the decrease in pain threshold, is in agreement with previous reports [69-71], further supporting involvement the sst2/p-p38 pathway in control of nociceptive thresholds.

## Discussion

Our understanding of pain signaling at the spinal level has been greatly advanced during the last decade, both with regard to anatomy, neurochemistry, circuitry and physiology [72-77]. Here we focus on the SST system. Our results provide further evidence that in mouse sst2A is a membrane-bound receptor expressed in a subpopulation of nociceptive DRG neurons and in local dorsal horn neurons. We show that a systematically administrated sst2 agonist, Oct, causes a rapid, transient receptor internalization in DRG neurons as well as counteracts nerve injury-induced pain behaviors in the SNI model, in parallel with attenuation of p-p38 upregulation. sst2A is also internalized in dorsal horn neurons by Oct after ith administration. In some DRG neurons sst2A and the Y1R are co-internalized after Oct stimulation *in vivo,* hypothetically forming a heterodimer. The sst2A is anterogradely transported and, in fact much more pronounced, also retrogradely, but in this case as a complex with SST, possibly carrying information to the soma. Our findings of sst2A in human DRGs and, abundantly, in spinal cord, suggest that similar mechanisms may operate in rodents and humans, and that targeting sst2A receptors may lead to novel treatment strategies for neuropathic pain.

### sst2A in the dorsal horn

Presence of sst2A^+^ cell bodies and a dense plexus of processes has been reported in the dorsal horn of mouse [24] and rat [43,44,78]. In rat sst2A is present in 13-15% of all neurons in laminae I and II, all are GABA^+^ and >80% are glycine^+^, i.e. inhibitory, and they are different from the NK1^+^[47,78] and MOR^+^[43,44] neurons. We here report that many sst2A^+^ neurons co-express nNOS, a few galanin, SST or NPYY1R, but none PKCgamma. The SST neurons are, on the other hand, glutamatergic, that is excitatory [79].

Here we show that sst2A is present both in cell bodies in the dorsal horn and in motoneurons in the human spinal cord. In both locations the receptor is mainly membrane-bound. In addition there is a dense plexus of processes in the superficial dorsal horn with a lower density in other layers. The distribution of SST in human DRGs and spinal cord has previously been described [80-83].

### Trafficking of sst2A

Trafficking of SST receptors has been studied [84-87]. Here we confirm that i.v. administration of Oct induce internalization of sst2A in DRG neurons [24]. Interestingly, in the latter study sst2A internalization was not observed after treatment with the pan-sst agonist pasireotide, which binds with high affinity to all SSTRs [88,89].

The sst2A is an endosomally recycled receptor [90,91]. In the DRG neurons the internalization was almost complete 1 hr after systemic Oct injection, partly back at the plasma membrane after 6 hours, and virtually completely after 24 hrs. Also Oct applied intrathecally induced distinct internalization in the superficial dorsal horn neurons, as well as in DRG neurons. Whether the latter is due to spread to the DRG cell bodies, or represents an Oct action on the central afferents in the dorsal horn, remains to be studied.

Here we show that the Y1R is co-localized with sst2A on the membrane of some DRG neurons. The sst2 agonist Oct induced a parallel internalization of sst2A and Y1R, supporting existence of a heterodimeric complex, a view that however at this point is hypothetical. In previous studies NPY or NPY agonists did not cause internalization of Y1R in rat DRGs (X. Zhang, Z.Q. Xu and T. Hökfelt unpublished observations). Previously, constitutive heterodimerization of sst2A/sst3 has been reported, the heteromers behaving like sst2A dimers, the sst3 being functionally inactivated [92]. Moreover, heterodimerization of sst2A and the μ-opioid receptor did not distinctly change ligand binding or coupling properties [93].

### Axonal transport of sst2A

sst2A is axonally transported in the sciatic nerve, accumulating around the lesion 10 hrs after a crush, the distal pile up, surprisingly, being much more pronounced, and sharply contrasting the strong proximal/modest distal CGRP accumulation. Interestingly, whereas on the proximal side SST^+^ and sst2A^+^ fibers were clearly separated from one another, as is expected in view of lack of coexistence in the DRG neuron cell bodies, the two markers appeared to coexist on the distal side. Thus, SST of peripheral origin may bind to sst2A, internalize and travel retrogradely as a SST/sst2A complex. This is supported by presence of sst2A^+^ nerve fibers in the skin, the distal pile up of exogenously infused SST-RFP and by the preferential, proximal SST accumulation in sst2-KO mice. The marked reduction of distal SST accumulation in the KO mice strongly suggests that the sst2A receptor is required for this pile up in control mice. This is similar to, e.g. nerve growth factor (NGF), and raises the possibility that the SST/sst2A complex represents a retrograde signal, the role of which has still to be defined. The origin of peripheral SST is potentially manifold: ‘endocrine’ SST in the blood, adjacent SST^+^ sensory branches and/or blood/immune cells [26].

### SST, its receptors and pain

There is now good evidence for a role of SST in pain signaling at the spinal level (see Introduction), involving DRG and local dorsal horn neurons. Both systems produce SST and certain SSTRs. In DRGs, and almost always in spinal cord, SST and sst2A are expressed in separate neuronal populations, excluding autoreceptor mechanisms.

Using unilateral SNI, a neuropathic pain model, the analgesic, sst2A-selective agonist Oct [33] significantly increased ipsilateral withdrawal threshold in the von Frey test, and decreased the withdrawal response-duration in the Pin-prick (noxious stimulus) and acetone (cold stimulus) tests for several hours. This is a peripheral action on DRG neurons, since Oct does not penetrate the blood–brain-barrier [20,48]. Moreover, Oct attenuates swelling and mechanical hyperalgesia in a mouse model of immune-mediated arthritis, an effect not seen in sst2A-KO mice [24], as well as capsaicin-induced nociceptor activity and nociceptive behavior *in vitro* and *in vivo*[94,95].

SST released from peripheral small diameter fibers can inhibit cross-excitation exerted by release of excitatory transmitters, such as glutamate and substance P, from adjacent primary afferent terminals [96,97], supported by our demonstration of sst2A expression in peripheral nerve terminals in the skin.

Ith SST has been reported to be pro-nociceptive [98-101], like substance P, even if high, probably toxic SST doses are anti-nociceptive [102,103]. SST causes outwards currents in lamina II sst2A^+^ neurons [69,104]. Yasaka et al. [19] have further clarified the molecular basis for the mainly pro-nociceptive effects exerted by SST in the spinal cord. They showed that SST produces outward currents in inhibitory GABA^+^/sst2^+^ interneurons, causing dis-inhibition. Thus, the SNI-induced upregulation of SST in DRG neurons shown here, and a hypothetical increased SST release, could contribute to increased pain signaling in the dorsal horn. However, we also show that ith Oct causes internalization of sst2A, and this may occur after endogenous SST release in the dorsal horn. This internalization should attenuate dis-inhibition and thus offer protection against pain. Finally, it is likely that sst2A is transported into sensory spinal afferents and here represent presynaptic inhibitory receptors, attenuating release of sensory, excitatory transmitters in the dorsal horn, like substance P, CGRP and glutamate, that is a further hypothetical mechanism involved in defense against pain.

It should be mentioned that the sst4 receptor is of interest in relation to pain, a view advanced in particularly by Szolcsányi and colleagues, based on transgenic mice and the use of an sst4-selective agonist, J-2156. Thus, this compound has analgesic effects in several pain models [31]. Moreover, sst4 KO mice exhibit an impaired defense against inflammation and hyperalgesia [30]. However, it is assumed that these sst4 not necessarily are located on neurons in DRGs or spinal cord [30]. Finally, as mentioned above, sst1 may also play a role in nociception based on its high proportion and significant changes in DRGs in response to nerve injury.

### NPY, Y1R and pain

The spinal NPY system has been implicated in pain transmission [105,106]. NPY is abundantly expressed in local dorsal horn neurons but under normal circumstances not in DRG neurons [107]. The Y1R is located both in rodent DRG and dorsal horn neurons [56,61,62]. The localization of both Y1R and sst2A on the cell membrane of DRG neurons may confer increased analgesia, since both sst2A and Y1R are anti-nociceptive [20,21,24,42,56,108-110]. To what extent co-internalization influences pain signaling remains to be analyzed. This said, it should be emphasized that the Y1R is expressed in a much larger DRG neuron population than sst2A.

### Signal transduction pathways

With regard to mechanism of action, we analyzed expression of p38 MAPK [63,64], a downstream molecule in the sst2 cascade [67,68]. Its phosphorylated form, p-p38, was strongly upregulated in the ipsilateral DRG after unilateral SNI, paralleling the decrease in pain threshold, in agreement with previous reports [69-71]. Oct, but not saline or Cyn154806, significantly reduced this upregulation, indicating that the sst2/p-p38 pathway may be involved in control of nociceptive thresholds.

## Conclusions

The present study reports presence of sst2A on the membrane of neurons in mouse and human DRGs and spinal dorsal horn. The sst2A agonist Oct causes internalization of the receptor both in mouse DRG and local dorsal horn neurons and has an anti-allodynic effect in a mouse model for neuropathic pain. Our findings suggest that peripheral sst2A may represent an attractive therapeutic target for treatment of neuropathic pain.

## Methods

### Animals

The experiments were performed on male C57BL/6 J Bommince mice (A/S Bomholtgaard, Ry, Denmark) weighing 25–28 g. Adult sst2 KO male mice (n = 10) with the C57BL/6 J background [111,112], and age- and sex-matched wild-type (WT) mice (n = 10) were also included, as well as three GAD-67-GFP knock-in mice [113]. Human ganglia and spinal cord were collected from a 48-year-old woman who died from stroke. The studies have been approved by the local Ethical Committee for animal experiments (Norra Stockholms djursförsöksetiska nämnd), and experiments on human DRGs were approved by a local Ethical Committee with written consent from the next of kin.

### Surgeries

Surgical procedures were performed under anesthesia with isoflurane (2%). Unilateral axotomy [114] and SNI [51,52] was made as described. Dorsal rhizotomy and sciatic nerve ligation were performed as previously described [115]. Survival times were 14 or 15 days. For analysis of intra-axonal transport, the sciatic nerve was ligated at mid-thigh level and animals exsanguinated after 10 hrs. Conjugated SST-Red fluorescent protein (RFP) was produced in bacteria as described (see below). The SST-RFP sample was concentrated and desalted by using Nanosep™ 3 K omega centrifugal device (Pall Corporation, Port Washington, NY). A single dose of desalted SST-RFP (48.9 mg/ml; 5 μl) was injected into the left hind paw with a Hamilton syringe, and the mice were exsanguinated 10 hrs or 3 days after injection.

To study sst2A trafficking, a single dose of each drug, i.e. Oct (1 μg/10 μl, i.v. or ith injection; dissolved in saline), Cyn154806 (0.2 μg/10 μl, i.v. or ith injection; dissolved in saline) or saline was administered to normal animals, and the tissues (DRGs and spinal cord) were collected 1 hr, 6 hrs or 24 hrs after injection. To study the effects of drugs on nociception, a single dose of each drug, i.e. Oct (40 μg/kg), Cyn154806 (6 mg/kg) or saline, was given i.p. 14 days after SNI. The behavioral tests were performed on SNI-treated animals 30 min after injection. The selection of dosage of Oct and Cyn154806 was based on previous studies [20,21,24,116-118].

### Production and characterisation of recombinant SST-mRFP protein

The genetic engineering procedure, microbial culturing and cell lysis were carried out as per the standard protocols. Briefly, his-tagged SST-mRFP, the DNA fragment encoding SST (AGCKNFFWKTFTSC) was reconstituted using a pair of primers 5′-TCATGGGTACCGGAGGTGGAGGTTCCGGAGGTGGAGGAT AAGAACTTC-3and 5′-ATGACAAGCTTATCAGCAGGATGTAAAGGTCTTCCAGAAGAAGTTCTTGCATCCAGCAG-3′ followed by fusing to mRFP [119] using a flexible peptide (Gly_4_Ser)_2_ linker. The DNA fragment was cloned into pQE-30 vector (QIAGEN, Hilden, Germany) [120], transfected into *E. coli* Origami™ B strains (Novagen®, Billerica, MA, USA) and cultured in Terrific Broth (TB), followed by lysis and Ni-NTA Superflow Cartridge purification assay*.* Sodium Dodecyl Sulfate Polyacrylamide Gel Electrophoresis was carried out to confirm that a well-defined band with a molecular weight of approximately 30 kDa, consistent with individual molecular masses of SST (1.63 kDa) and SST-mRFP (29.73 kDa). The activity and potency of the produced SST-mRFP was evaluated by using the membrane potential assay and mouse AtT-20 neuroblastoma cell line (ATCC, VA,USA), as detailed [120]. The activity of as produced SST-mRFP was estimated in terms of pEC50 value and found to be 5.5. SST-mRFP appeared to be 100 times less potent than SST.

### Behavioral tests

von Frey filament, Pin-prick and acetone stimuli were applied to the lateral and medial plantar surface of mice hind paws as previously described [115].

### Immunohistochemistry

Mice were perfused with 4% paraformaldehyde and 0.4% picric acid in 0.16 M phosphate buffer (pH 7.2; 37°C) as described in [121]. The human DRGs and spinal cord were immersion-fixed for 4 hrs in ice-cold fixative. Tissues were cut in a cryostat (Microm, Heidelberg, Germany) at 14 μm (mouse and human DRGs) or 20 μm (spinal cord) thickness. Immunostaing procedures with tyramide signal amplification system were performed as previously described [122]. Primary antisera/antibodies used were monoclonal anti-sst2 antibody (clone UMB-1) [24,123], rabbit anti-CGRP [124], NPYY1R [61], NPYY2R [125], rabbit anti-galanin (gift from late Drs J. Walsh and H. Wong), SST (gift from Dr. A. Benoit, Montreal General Hospital, Montreal, Canada), PKCgamma (Santa Cruz Biotechnologies, Dallas, TX) and ATF3 (Santa Cruz Biotechnologies), sheep anti-nNOS [126] and chicken anti-β-gal (1:1,000) (Abcam, Cambridge, UK), In addition, a group of sst2A-labeled sections was incubated with the IB4 from Griffonia simplicifolia I (GSA I; IB4; 2.5 μg/ml; Vector Laboratories, Burlingame, CA) [127].

### Image analysis and quantification

Specimens were analyzed in a Bio-Rad Radiance Plus confocal scanning microscope (Bio-Rad, Hemel, Hempstead, UK) installed on a Nikon Eclipse E 600 fluorescence microscope (Nikon, Tokyo, Japan) and, in some experiments, an LSM 700 confocal microscope (Zeiss, Oberkochen, Germany) as in previous work [122]. The percentage of ^+^NPs in DRGs was obtained as described [115]. Four to 8 sections of each DRG from 5 animals in each group were included in the analysis. The size distribution of ^+^NPs with a visible nucleus was measured using the Nikon Eclipse E 600 fluorescence microscope with Wasabi Image Software. The ^+^NPs were divided into small, medium-sized and large according to earlier studies [53]. The relative fluorescence levels (intensity) of sst2A-like immunoreactivity (LI) in DRGs before and after nerve injury were measured as described [115] using a Sarastro 1000 confocal laser-scanning system (Molecular Dynamics, Sunnyvale, Calif., USA).

### Real-time quantitative PCR

Quantitative PCR (qPCR) reactions were performed with iQ SYBR green Supermix on a Bio-Rad MyIQ thermal cycler (BioRad). Preparation of samples and calculations were performed as described [122]. Each sample was run in triplicate to avoid processing-related deviations. Analysis was performed in Prism 6 software. The following primers were used: (1) sst1 forward primer 5′-TGG TGG GCT TCG TCT TAT-3′; (2) reverse primer 5′-GAT GAC AGA CAA CTG GCT CA-3′; (3) sst2 forward primer 5′-GGC GTG GTA CAC AGG TTT C-3′; (4) reverse primer 5′-GAA GAC AGC CAC TAC GAT GG-3′ [128].

### Western blot analysis

Sciatic nerve samples from operated animals for western blot were processed as described [122]. Laemmeli buffer containing sample protein was separated on 10% SDS-PAGE gel and transferred to polyvinylidene fluoride membranes (Millipore, Hemel, Hempstead, UK), which were incubated with the antibody against sst2A (1:400). After incubation with secondary antibody, the membranes were developed by ECL solution (Amersham Biosciences, Piscataway, NJ) and exposed to X-ray film (NEN PerkinElmer, Waltham, MA). GAPDH (anti-rabbit, 1:5,000 in 5% BSA; Cell Signaling, Beverly, MA) was chosen as the loading control.

### In situ hybridization

Experiment was carried out essentially as described previously [129], using oligonucleotide probes for sst2 [130] and SST [131]. A mixture of two oligonucleotide probes complementary to nucleotide sequences of the human sst2 was purchased from CyberGene (Stockholm, Sweden) or MWG Biotech (Ebersberg, Germany): (1) 5′ATT TGT CCT GCT TGT CAC TCC GCT C3′ and (2) 5′ TAT TGG CTT CAC GGT AAG TCC ATT TCT GCG 3′. The 33P-dATP-labeled sections were exposed for 6 weeks after dipping with emulsion solution. Some developed sections were counterstained with cresyl violet and mounted with Entellan (Merck, Darmstadt, Germany). Photographs were taken with a Nikon Coolpix 5000 digital camera (Nikon).

### Statistical analyses

Data are expressed as mean ± SEM. Differences between groups were compared using unpaired or paired Student’s *t* test (two groups). Some data (n = 4 samples) were also tested by the non-parametric Kruskal–Wallis test and Mann–Whitney test (STATISTICA, Version 10). A *P* value less than 0.05 was regarded as being significant.

## Abbreviations

β-gal: β-galactosidase; CGRP: Calcitonin gene-related peptide; DRG: Dorsal root ganglion; GAD-67: Glutamic acid decarboxylase 67; IB4: Isolectin ib4; IR: Immunoreactive; L: Lumbar; LI: Like immunoreactivity; NP: Neuron profile; NPY: Neuropeptide tyrosine (Y); NPYY1R and -2R: Neuropeptide tyrosine (Y) receptor 1 and 2; nNOS: Neuronal nitric oxide synthase; Oct: Octreotide; p38 MAPK: The mitogen-activated protein kinase p38; PGP 9.5: Protein gene product 9.5; PKC gamma: Protein kinase C gamma; RFP: Red fluorescent protein; SST: Somatostatin; sst1 and -2: Somatostatin receptor 1 and 2.

## Competing interests

The authors declare that they have no competing interest.

## Authors’ contributions

TJSS and TH designed the study; TJSS, QX, MDZ, SB, YKL, LG, and SMD carried out the experiments; AJ, SS, SMD and AVZ supplied reagents; TJSS, QX, MDZ, KF and TH wrote the paper. All authors read and approved the final manuscript.
